# The time-course of protection of the RTS,S vaccine against malaria infections and clinical disease

**DOI:** 10.1186/s12936-015-0969-8

**Published:** 2015-11-04

**Authors:** Melissa A. Penny, Peter Pemberton-Ross, Thomas A. Smith

**Affiliations:** Department of Epidemiology and Public Health, Swiss Tropical and Public Health Institute, 4051 Basel, Switzerland; University of Basel, Petersplatz 1, Basel, Switzerland

**Keywords:** Malaria, Vaccine, RTS,S, Model, Simulation

## Abstract

**Background:**

Recent publications have reported follow-up of the RTS,S/AS01 malaria vaccine candidate Phase III trials at 11 African sites for 32 months (or longer). This includes site- and time-specific estimates of incidence and efficacy against clinical disease with four different vaccination schedules. These data allow estimation of the time-course of protection against infection associated with two different ages of vaccination, both with and without a booster dose.

**Methods:**

Using an ensemble of individual-based stochastic models, each trial cohort in the Phase III trial was simulated assuming many different hypothetical profiles for the vaccine efficacy against infection in time, for both the primary course and boosting dose and including the potential for either exponential or non-exponential decay. The underlying profile of protection was determined by Bayesian fitting of these model predictions to the site- and time-specific incidence of clinical malaria over 32 months (or longer) of follow-up. Using the same stochastic models, projections of clinical efficacy in each of the sites were modelled and compared to available observed trial data.

**Results:**

The initial protection of RTS,S immediately following three doses is estimated as providing an efficacy against infection of 65 % (when immunizing infants aged 6–12 weeks old) and 91 % (immunizing children aged 5–17 months old at first vaccination). This protection decays relatively rapidly, with an approximately exponential decay for the 6–12 weeks old cohort (with a half-life of 7.2 months); for the 5–17 months old cohort a biphasic decay with a similar half-life is predicted, with an initial rapid decay followed by a slower decay. The boosting dose was estimated to return protection to an efficacy against infection of 50–55 % for both cohorts. Estimates of clinical efficacy by trial site are consistent with those reported in the trial for all cohorts.

**Conclusions:**

The site- and time-specific clinical observations from the RTS,S/AS01 trial data allowed a reasonably precise estimation of the underlying vaccine protection against infection which is consistent with common underlying efficacy and decay rates across the trial sites. This calibration suggests that the decay in efficacy against clinical disease is more rapid than that against infection because of age-shifts in the incidence of disease. The dynamical models predict that clinical effectiveness will continue to decay and that likely effects beyond the time-scale of the trial will be small.

**Electronic supplementary material:**

The online version of this article (doi:10.1186/s12936-015-0969-8) contains supplementary material, which is available to authorized users.

## Background

Malaria continues to cause significant burden and mortality worldwide with an estimated 580,000 deaths each year [1]. This represents large reductions in malaria mortality from 2000 levels due to large-scale roll-out of insecticide-treated nets and improved access to malaria treatment. However, new tools, such as malaria vaccines, may play a role in further reducing burden and preventing infection. The final results of Phase III clinical trials of malaria vaccine RTS,S were recently published [2], demonstrating efficacy against clinical disease in the two age groups that were vaccinated in 11 trial sites across malaria-endemic Africa (in the presence of high insecticide-treated net (ITN) usage and high quality access to care). The observed proportionate reduction in incidence of clinical malaria comparing the vaccinated and the control group, called here the efficacy against clinical disease, declined over time from 60 % during the first 6 months to 16 % during months 21–32 of follow-up in children aged 5–17 months old; in infants aged 6–12 weeks, the efficacy declined from 44 to 8 % during the same periods. For the entire follow-up period of 32 months, observed efficacy against clinical malaria was 35 % (95 % CI 31–40 %) in children and 20 % (95 % CI 14–27 %) in infants [2]. A boosting dose, given 18 months after the primary course of three doses, extended protection but not to the level seen in the 6 months following the primary course. Efficacy against clinical malaria in the booster cohorts was 44 % (95 % CI 40–48 %) in children and 28 % (95 % CI 22–33 %) in infants over the entire follow-up period of 32 months, but in months 21–32 of the trial (immediately after boosting) vaccine efficacy of 38 % for children and 27 % for infants was observed.

This trial provides estimates of efficacy against clinical malaria, but does not provide direct estimates of the underlying protection against infection or of the dynamics of protection over time. Mathematical models of malaria dynamics are needed to infer the underlying level of protection against infection, allowing for the dynamics of pathogenesis and of the acquisition of immunity. When calibrated with trial data, such models can be used to elucidate how setting specific factors (such as the level of exposure to malaria infection) modify these dynamics to give rise to the observed clinical efficacy.

RTS,S acts by inducing an immune response against the circumsporozoite protein (CSP) of the sporozoites of the *Plasmodium* parasite. When high immune responses are achieved, this reduces the chances of liver infection and subsequent blood-stage infection, and hence clinical malaria. Partial immunity to the different life stages of the malaria parasite within-host is induced naturally by repeated malaria infection, and thus non-vaccinated individuals more rapidly acquire immunity to the blood-stage parasites causing clinical illness. RTS,S, or indeed any other partially protective malaria infection blocking intervention aimed at infants and young children, will thus give rise to age shifts of burden and increased susceptibility to infection in older children [3, 4].

Previous analysis from the 18-month follow-up of RTS,S Phase III results [5] predicted that RTS,S has a high initial efficacy against infection (greater than 80 % in children vaccinated between five and 17 months and 65 % in infants vaccinated between 6 and 12 weeks), with an exponential half-life of decay of efficacy against infection of around 1 year [6]. However, the length of follow-up from the trial results was too short to determine if exponential decay was the most likely profile of decay of protection. Analysis of RTS,S Phase II antibody data indicated that biphasic exponential dynamics, with a quick decay followed by a long decay, fit the data better than a single exponential [7]. More recently, observations of incidence and efficacy in the boosting cohorts have become available and can be used to determine the underlying protection against infection offered by boosting doses [2].

This paper extends previous analysis [6] using the final published results of RTS,S in Phase III studies [2] to determine the time course of protection of RTS,S following a primary course of three doses in infants and children and following a boosting dose at 18 months post third dose. The temporal profile of RTS,S efficacy against infection is estimated using the malaria epidemiology microsimulation platform OpenMalaria [8], the observed incidence in the vaccinated and control cohorts and Bayesian Markov Chain Monte Carlo (MCMC) methods. This further allows use of OpenMalaria simulations to project the efficacy against clinical and severe disease in each of the trial sites for follow-up longer than that so far published.

## Methods

### RTS,S trial cohorts and data

The number of infants or children in the intention-to-treat analysis of the Phase III RTS,S trial, along with the recorded number of cases of clinical malaria, primary case definition, at 3 monthly time points per trial site, were used in this study. All data were previously published in [2].

### Vaccine trial simulations of RTS,S Phase III clinical trials with OpenMalaria

OpenMalaria is an individual-based, stochastic model of malaria epidemiology and control [8, 9] that includes model components of malaria in mosquitoes [10], dynamics of infection to humans [11], blood-stage parasite densities [12], infectiousness to mosquitoes [13], incidence of morbidity including severe, and hospitalization and mortality [14, 15]. Pre-erythrocytic and blood-stage immunity comprise separate sub-models, with blood-stage immunity predominating as infection-blocking immunity occurs only in those with very high cumulative exposure [12]. The ensemble of six model variants, described in Table 1, include different assumptions for decay of natural immunity, greater within-host variability between infection and entomological exposure and heterogeneity [8].

**Table 1:** Summary of simulations: variables and levels

Variable	Details and levels simulated
Vaccination: infant cohort (EPI cohort)	6, 10, 14 weeks; booster at 21 months
Vaccination: children cohort (5–17 months cohort)	Ages between 5–17 months first dose, and for 3rd dose 8–20 months; booster at 26–38 months
Model variants [8]	1. R0000 Base model 2. R0068 Heterogeneity in transmission: within-host variability 3. R0131 Immunity decay in effective cumulative exposure 4. R0132 Immunity decay in immune proxies 5. R0133 Immunity decay in both immune proxies and effective cumulative exposure 6. R0670 Heterogeneity in susceptibility to co-morbidity
EIR	0.1^a^, 1, 2, 4, 8, 16, 64, 256
Access to uncomplicated case management (%)^b^	0, 5, 40
Access inpatient care for severe cases (%)^c^	0, 100
Vaccination coverage^d^	0, 100
Initial efficacy against infection of third dose (%)	30, 60, 80, 100
Half-life (years)	0.5, 1, 3, 5
Initial efficacy against infection of boosting dose (%)^e^	Third dose efficacy, 30, 100
Weibull decay shape parameter (*k*)	*k* = 1 (exponential) *k* = 0.5 (bi-phasic, quick decay followed by slow decay) *k* = 3 (slow decay, followed by quick decay)

^a^EIR of 0.1 was not simulated, predictions for this level are taken as 10 % of EIR 1

^b^Probability of access to treatment for uncomplicated disease during a 5-day period (for mapping onto rates of access estimated from survey data see [6, 31]

^c^Probability of access to hospital care (or equivalent) for severe disease during any 5-day period

^d^For each of the four delivery schedules

^e^This represents the absolute efficacy against infection achieved by the addition of a booster doses and not a percentage of the third dose

Each arm of the vaccine trial was explicitly simulated with an OpenMalaria ensemble of six models [8] (Table 1). Similar to previous analysis, predictions of clinical trial outcomes were calculated via weighted averages of a large number of simulations [6]. The simulations covered a wide range of vaccine profile characteristics, deployed across a range of health system and transmission settings of the trial sites. The weights applied to each simulation are dependent on the trial-specific data and on vaccine properties for fitting.

Six databases of simulations from OpenMalaria were created, two for baseline (control cohorts 6–12 weeks and 5–17 months) and four for vaccine impact predictions (boost and non-boosting schedules for 6–12 weeks and 5–17 months) over a range of assumptions on transmission exposures [entomological inoculation rates (EIR)], timing of vaccination due to seasonality profile, range of access to effective treatment for uncomplicated cases, and for many hypothetical vaccine efficacy profiles (initial efficacy against infection, decay shape and boosting efficacy against infection, with boosting efficacy defined as the overall efficacy reached by an additional fourth vaccine dose, not incremental efficacy addition to the primary course). Each database was a complete factorial combination of all levels of each variable listed in Table 1, with a database constructed for each of the 6–12 weeks cohorts with and without booster, 5–17 months cohorts with and without booster, and the no-vaccine cohorts. This resulted in over 500,000 simulations when replicates with different stochastic initializations were counted. Outputs from the simulations were recorded at 3-monthly intervals, for 3-monthly age groups, with vaccination occurring at the ages specified in Table 1. For each simulation the numbers of uncomplicated cases, severe cases, direct malaria deaths, indirect malaria deaths, sequelae events, first-line, second-line and third-line treatments given, hospitalized cases resulting in recovery, hospitalized cases that resulted in sequelae and hospitalized cases that resulted in death were recorded, to allow incidence calculations to compare to trial data.

Predictions of incidence for each cohort and clinical efficacy in time per vaccination schedule in each trial site were obtained via weighted averages over all simulations in the appropriate database (see [6] for full methods). Predictions comprise mean weighted averages with an accompanying range constructed from the 95 % prediction interval over the weighted averages for all models and stochastic runs, with each model variant equally weighted. The prediction intervals thus capture both structural and stochastic uncertainty.

Trial-specific parameter weights for transmission and access to care were pre-determined or estimated by fitting to the trial data. Separate estimates were made for each trial site of the levels of access to treatment and of the exposure (EIR). This entailed fitting to the control incidence disaggregated into 3-monthly time intervals and the control cohort at baseline in the trial site.

The posterior densities of the prevalence in 2–10 years olds (*Pf*PR_2–10_), estimated from a geostatistical model at 5 × 5 km resolution by the Malaria Atlas Project 2010 (MAP) [16], were used to capture effects of within-site heterogeneity in transmission. A previously published algorithm using OpenMalaria [17] was used to derive distributions of EIR for each trial site, as functions of the *Pf*PR_2–10_ data, access to treatment data for the same geographic area estimated from demographic and health surveys (DHS), and the population of each pixel derived from the demographic population surfaces [18] (Table 2). For simulation of the trial, these EIR distributions were scaled so that the average prevalence predicted by OpenMalaria matched the baseline prevalence measured in each trial site and each site was then treated as having a mixture of transmission intensities corresponding to this scaled EIR distribution. In the trial simulations, access to care for each site was assigned the value required to recover the incidence of diagnosed clinical malaria reported in the control (non-vaccine) arms.

**Table 2:** Estimated characteristics of the sites

Site	Country	Median EIR derived from MAP *Pf*PR_2–10_	Median EIR adjusted for trial prevalence and incidence in the control	DHS estimate of access to effective treatment	Estimated access to effective treatment in trial
Kilifi	Kenya	1.1	0.15	45.0	53.9
Korogwe	Tanzania	2.0	0.12	49.2	35.3
Manhica	Mozambique	2.4	0.15	35.8	46.7
Lambarene	Gabon	4.3	0.18	16.1	60.8
Bagamoyo	Tanzania	2.9	0.23	55.4	54.1
Lilongwe	Malawi	6.3	0.44	43.3	52.6
Agogo	Ghana	6.3	2.1	42.5	63.5
Kombewa	Kenya	5.2	8.7	48.8	59.3
Kintampo	Ghana	19.5	13.5	40.5	53.9
Nanoro	Burkina Faso	89.2	75.6	37.4	39.4
Siaya	Kenya	34.5	86.6	48.8	50.9

### Vaccine efficacy against infection and decay

The action of RTS,S (or other pre-erythrocytic vaccines) in OpenMalaria is to prevent new infections, with the proportion of blood-stage infections averted referred to as vaccine efficacy against infection. This is different from the efficacy in averting clinical episodes as reported in the Phase III clinical trials. The time course of efficacy against infection can be described by several possible decay functions in OpenMalaria [19]. Different Weibull decay function curves are investigated for describing the waning protection of RTS,S by an initial value of the efficacy against infection $\varepsilon_{0}$, a half-life *L*, and a shape parameter, *k*. The Weibull decay function for efficacy against infection, *ɛ*(*t*), at time *t*, takes the.

$$\varepsilon \left( t \right) = \varepsilon_{0} { \exp }\left( {\frac{{ - \left( {log2} \right)^{1/k} t}}{{L^{k} }}} \right).$$

When *k* is 1, an exponential decay of efficacy against infection is obtained. For *k* < 1, the initial decay is faster than exponential and then slower than exponential after the time equivalent to half-life is reached, this is similar to a bi-phasic like decay, with a sharp decline (quick decay) in efficacy followed by a slower decay. For *k* > 1, the curves is a slow decay of efficacy against infection until the time equivalent to half-life *L*, and then a much faster decay.

### Determining vaccine properties from Phase III clinical trial data

Using Bayesian MCMC methods, comparing simulated incidence and Phase III trial incidences, the efficacy profile for each cohort was determined in terms of initial efficacy against infection following the primary schedule of three doses. This profile comprises the half-life of decay of efficacy against infection, the shape parameter describing the waning profile, and the efficacy against infection following a boosting dose. Models were simultaneously fit to the control and vaccinated incidence from each trial site for the primary case definition, 3-monthly or 6-monthly aggregated intention-to-treat (ITT). Data from the Kilifi and Manhica were not used for fitting of vaccine properties because of challenges related to missing data: instead, the predictions for these sites provided an out-of-sample check on the performance of the fitting algorithm.

As previously described in [6], a Bayesian MCMC approach was used to estimate vaccine properties, site-specific access to care, and the extent of within-site variation in clinical disease (number of episodes per individual for a defined time period). The observed clinical data (disease rates in the control and vaccinated groups at each time point) are assumed to be normally distributed about the logarithm of their predicted values for a given set of parameters. i.e.:

$${ \log }\left( {{\text{Y}}_{{{\text{t}},{\text{i}}}} } \right)|{{\theta }},{{\sigma }}_{i} \sim {\text{Normal}}\left( {{ \log }\left( { {\hat{{\mu }}}_{t,i} \left( {{\theta }} \right)} \right),{{\sigma }}_{i} } \right) ,$$

where Y_t,i_ is the observed disease rate (for control or vaccinated) at time *t* and trial site *i*, ${\hat{{\mu }}}_{t,i}$ is the weighted model prediction for the equivalent outcome at time *t* and site *i*, *θ* represents the parameters being fitted (vaccine properties and access to care), σ_*i*_ is the standard-deviation for trial site *i*, ${\hat{{\mu }}}_{t,i}$, is the model prediction, obtained as the weighted average of simulated disease rates from the databases of vaccine cohort predictions from OpenMalaria (Table 1). The weights for site *i* were assigned to give the pre-determined distribution of EIR values representing malaria exposure in that site while an MCMC algorithm was used to sample parameters and estimate the weights for efficacy, half-life, decay shape and the access to care parameter.

Vaccine parameters for each age of vaccination cohort were fitted separately, so that independent parameters for the time course of vaccine efficacy against infection for the primary schedule were obtained. Vaccine parameters were assumed to take the same value for each site. To test robustness of the models, models were first fitted to aggregated 6-monthly observed incidence data per site and subsequently to the 3-monthly aggregated data. Parameterizating was also done assuming both exponential decay (Weibull decay function shape parameter *k* = 1) and also allowing the decay shape parameter to vary.

Two MCMC chains with very different initial conditions for efficacy, access to care, decay shape and half-life were used for each fitted model. Uniform [0, 1] priors were used for all parameters except the half-life of vaccine efficacy decay, which was assigned a Uniform [0, 5] years prior, with non-informative (uniform) priors for all parameters. Posterior distributions were sampled for each of the fitted parameters (6–12 weeks initial efficacy against infection, 5–17 months initial efficacy against infection, vaccine half-life of decay, decay shape parameter, within site variation against clinical disease and site-specific access to care).

Following determination of the vaccine efficacy profile of the primary course, the boost efficacy was obtained via Bayesian MCMC in a similar manner. Only the initial efficacy against infection as a result of a boosting dose was fitted, assuming the same decay shape as for the primary course (exponential or best fit when fitting Weibull decay parameter *k*).

### Predictions of clinical disease efficacy beyond the trial follow-up

Clinical efficacy beyond the trial follow-up of 32 months was projected using the same model weighting (and hence final vaccine profile) as estimated from the trial data.

## Results

### Characteristics of trial sites

There was considerable variation between the sites in average transmission levels as estimated from the MAP surfaces, with substantial local heterogeneity within each site (Table 2). However, the baseline EIR in each site was generally lower than the prediction from MAP, as well as spanning a smaller range across the sites than when estimated from MAP. Similarly, the estimated access to care derived from the DHS was lower than the estimates derived by comparing model predictions of incidence with recorded events in the control arm of the trial (Table 2) [6].

We conjecture that this difference arises from better health care service provision in the study sites, than neighbouring areas. Both MAP *Pf*PR_2-10_ and DHS-based estimates borrow information from adjacent geographical areas, and the sites were necessarily centred around high-quality health facilities with linked research infrastructure, and thus have unusually good access to both preventive and curative interventions.

### RTS,S vaccine efficacy against infection, time course for primary schedule of three doses

**Fig. 1:**
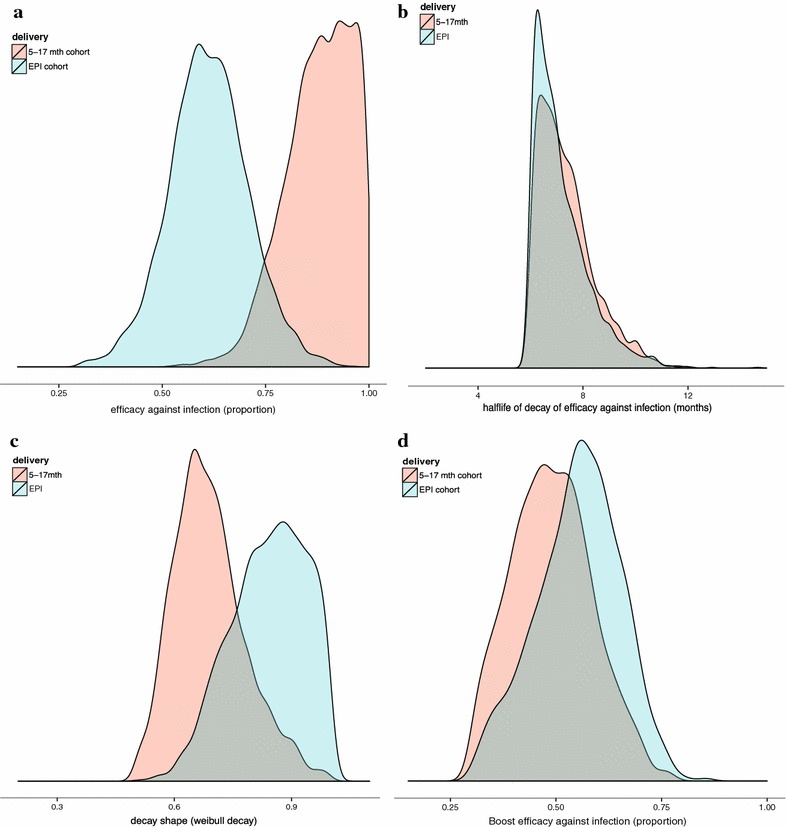
Posterior distributions of parameters for vaccine efficacy profiles (assuming Weibull decay function, fitted to trial data at 3-monthly periods). *Colour green* indicates 6–12 weeks cohort, and pink the 5–17 months cohort. **a** Vaccine initial efficacy against infection; **b** half-life of decay in efficacy against infection; **c** Weibull decay function shape parameters; **d** boost efficacy against infection

**Fig. 2:**
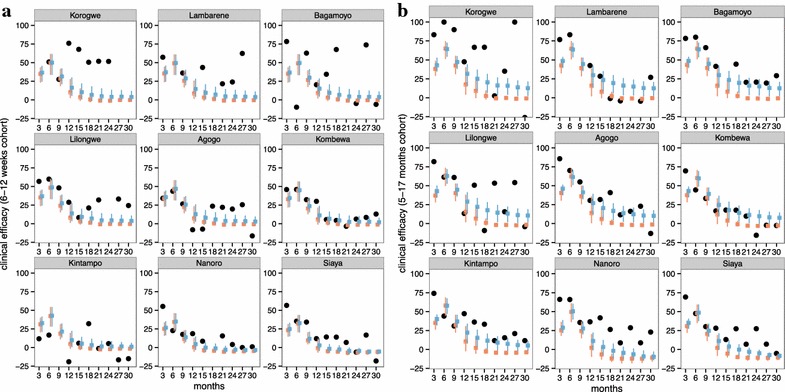
Observed and predicted efficacy against clinical disease for 3-monthly periods. Field and predicted estimates of clinical efficacy at each 3-month follow-up for **a** the 6–12 weeks without booster; **b** 5–17 months cohort without booster, by the 9 trial site used in the fitting (excludes Kilifi and Manhica). Reported efficacy (mean and 95 % CI) in the trial site is indicated by *black circles*. Prediction estimates (mean and 95 % prediction intervals) are shown in *colour* for *different fitted models*, *orange* assuming exponential decay and *blue* fitting for decay shape

Regardless of assumptions concerning the functional form of decay of efficacy, the initial protection of the RTS,S vaccine in children 5–17 months is estimated to be high (similar to previous analysis). Fitting to 3-monthly incidence data and allowing decay to be either exponential or non-exponential the estimated efficacy against infection immediately following third dose was 91.1 (95 % CI 74.5–99.7 %) for children vaccinated between 5 and 17 months at first dose (Table 3; Fig. 1a). In infants vaccinated 6–12 weeks the initial efficacy against infection was 64.9 % (95 % CI 44.0–83.2 %) (Table 3; Fig. 1a). Results were similar when fitting to 6-monthly decay (Additional file 1: Table S1). Models were fit to the clinical incidence in both control and vaccinated cohorts (results shown in Additional file 1: Figures S1, S2, S3, S4), with calculated vaccine efficacy for the best fitted models assuming exponential and Weibull decay are shown in Fig. 2.

**Table 3:** Best-fitted vaccine efficacy profiles for the 6–12 weeks and 5–17 months cohorts when fitting to 3-month incidence data from the RTS,S Phase III trial

Cohort	Initial efficacy against infection at completion of 3rd dose (%)	Half-life of efficacy against infection (months)	Decay (Weibull decay shape parameter)	Boosting efficacy against infection at 4th dose (%)
Exponential decay				
6–12 weeks	57.5 (95 % CI 40.1–71.2)	7.4 (95 % CI 6.1–10.4)	Exponential	48.5 (95 % CI 32.8–64.3)
5–17 months	72.5 (95 % CI 57.7–83.7)	7.9 (95 % CI 6.1–11.0)	Exponential	39.2 (95 % CI 30.6–53.4)
Weibull decay				
6–12 weeks	64.9 (95 % CI 44.0–83.2)	7.2 (95 % CI 6.0–9.8)	0.84 (95 % CI 0.64–0.99)	55.2 (95 % CI 34.5–73.1)
5–17 months	91.1 (95 % CI 74.5–99.7)	7.32 (95 % CI 6.0–10.0)	0.69 (95 % CI 0.54–0.9)	49 (95 % CI 32–68.6)

Posterior distributions described by mean and 95 % credible interval

Assuming exponential decay, the half-life of protection was approximately 7–8 months for both cohorts (Table 3; Fig. 1b and Additional file 1: Table S1). When allowing decay to be non-exponential, half-life of decay against infection was also around 7 months (95 % CI 6–10 months for both cohorts), with decay shapes for the 5–17 months cohorts *k* = 0.69 (95 % CI 0.54–0.9) indicating a quick decay followed by a longer decay. For the 6–12 weeks cohort the prediction is closer to exponential *k* = 0.84 (95 % CI 0.64–0.99).

### Boosting dose efficacy against infection

The efficacy against infection of the single boosting dose given 18 months following third dose was estimated to be lower than that immediately following the final dose of the primary course, with estimates of 49 % (95 % CI 32–68.6 %) for children 5–17 months and 55.2 % (95 % CI 34.5–73.1 %) for infants 6–12 weeks (Table 3; Fig. 1d). These estimates assume that the half-life and decay of the boosting dose were the same as fitted for the primary course.

### Predictions of efficacy against clinical disease

**Fig. 3:**
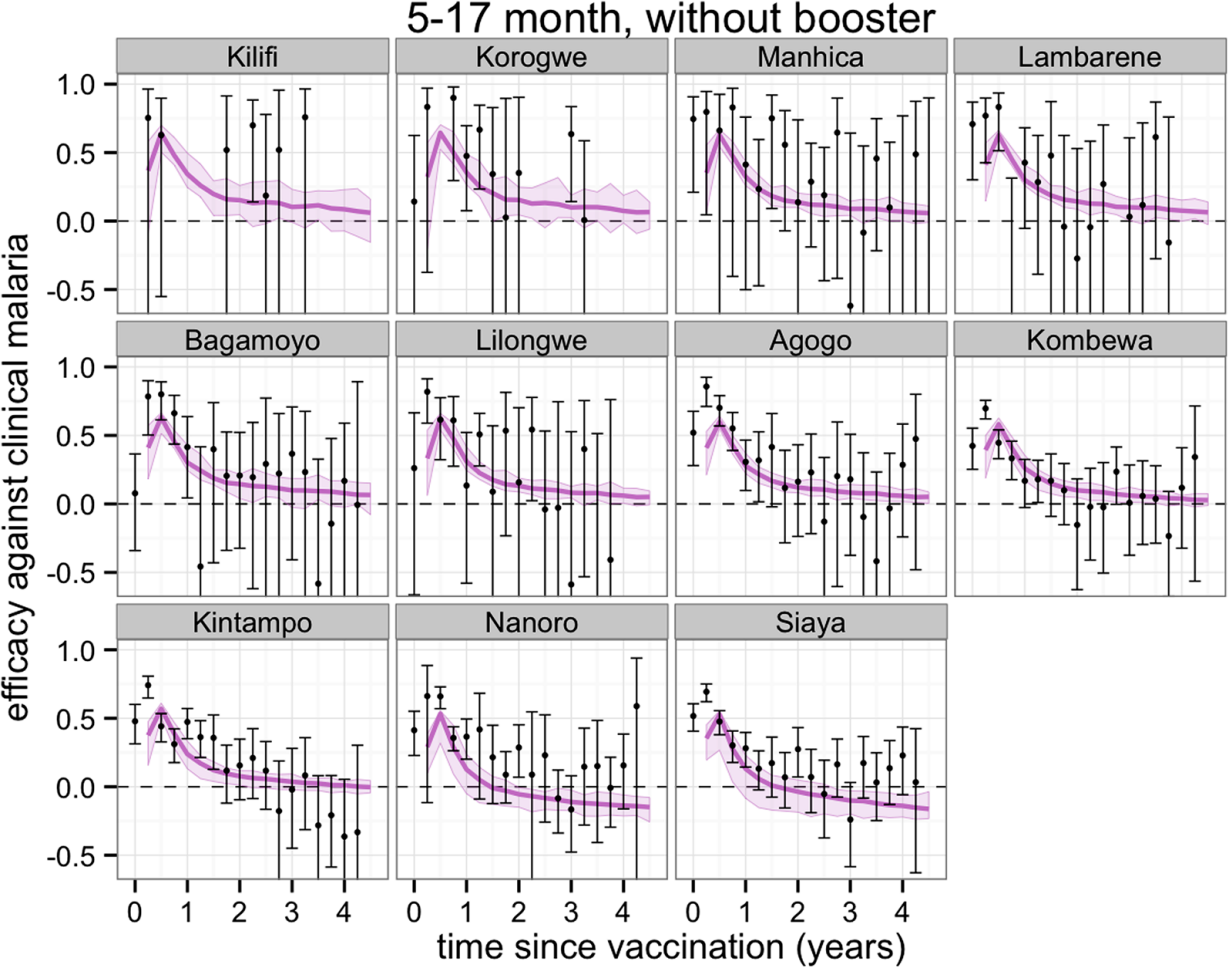
Predicted and observed efficacy against clinical disease by 3-monthly periods by trial site for the 5–17 months cohort using best-fitted vaccine profile. Reported efficacy (mean and 95 % CI) in the trial site is indicated by *black circles*. Prediction estimates in *purple* (median and 95 % CI)

**Fig. 4:**
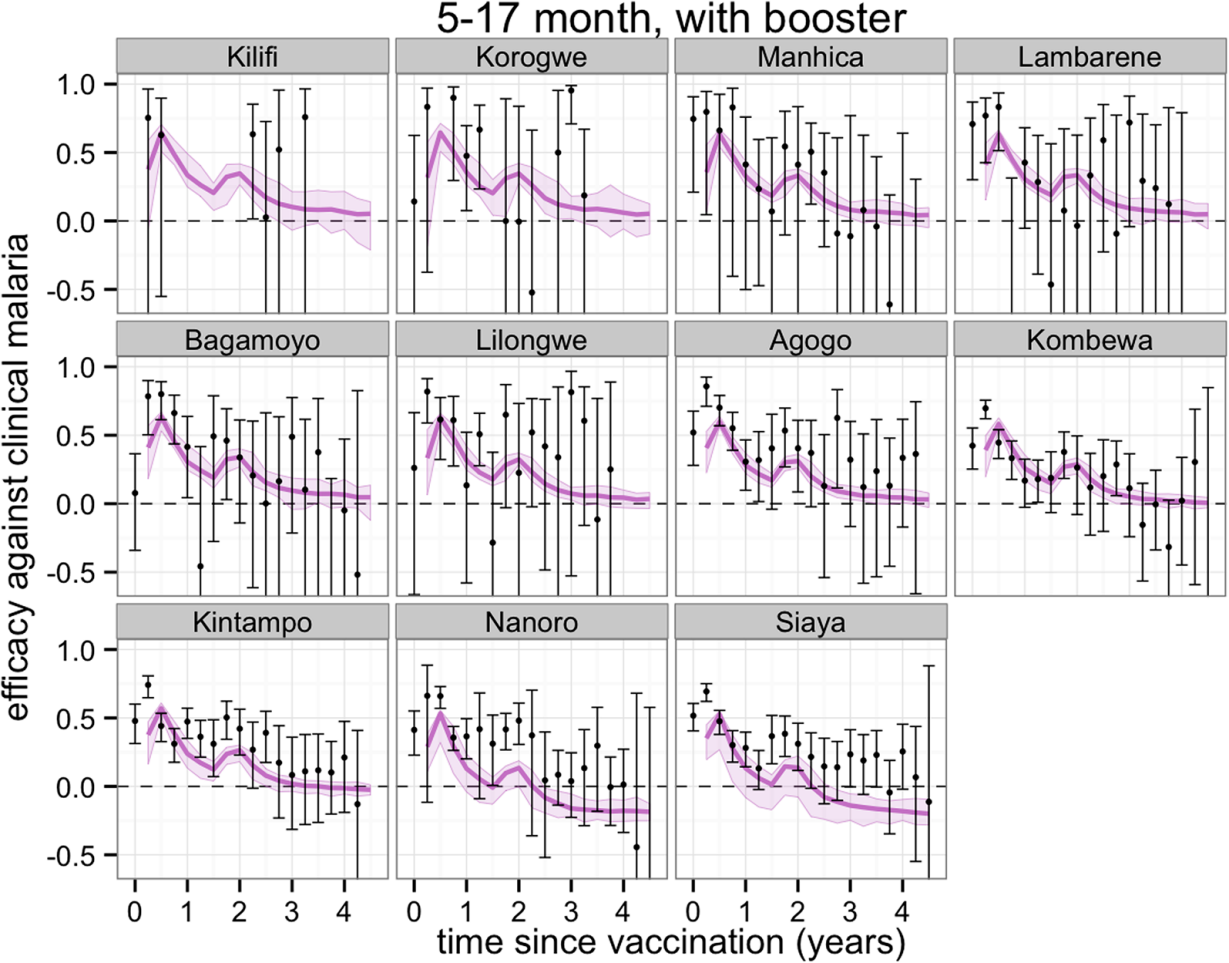
Predicted and observed efficacy against clinical disease by 3-monthly periods by trial site for the 5–17 months cohort with booster using best-fitted vaccine profile. Reported efficacy (mean and 95 % CI) in the trial site is indicated by *black circles*. Prediction estimates in *purple* (median and 95 % CI)

**Fig. 5:**
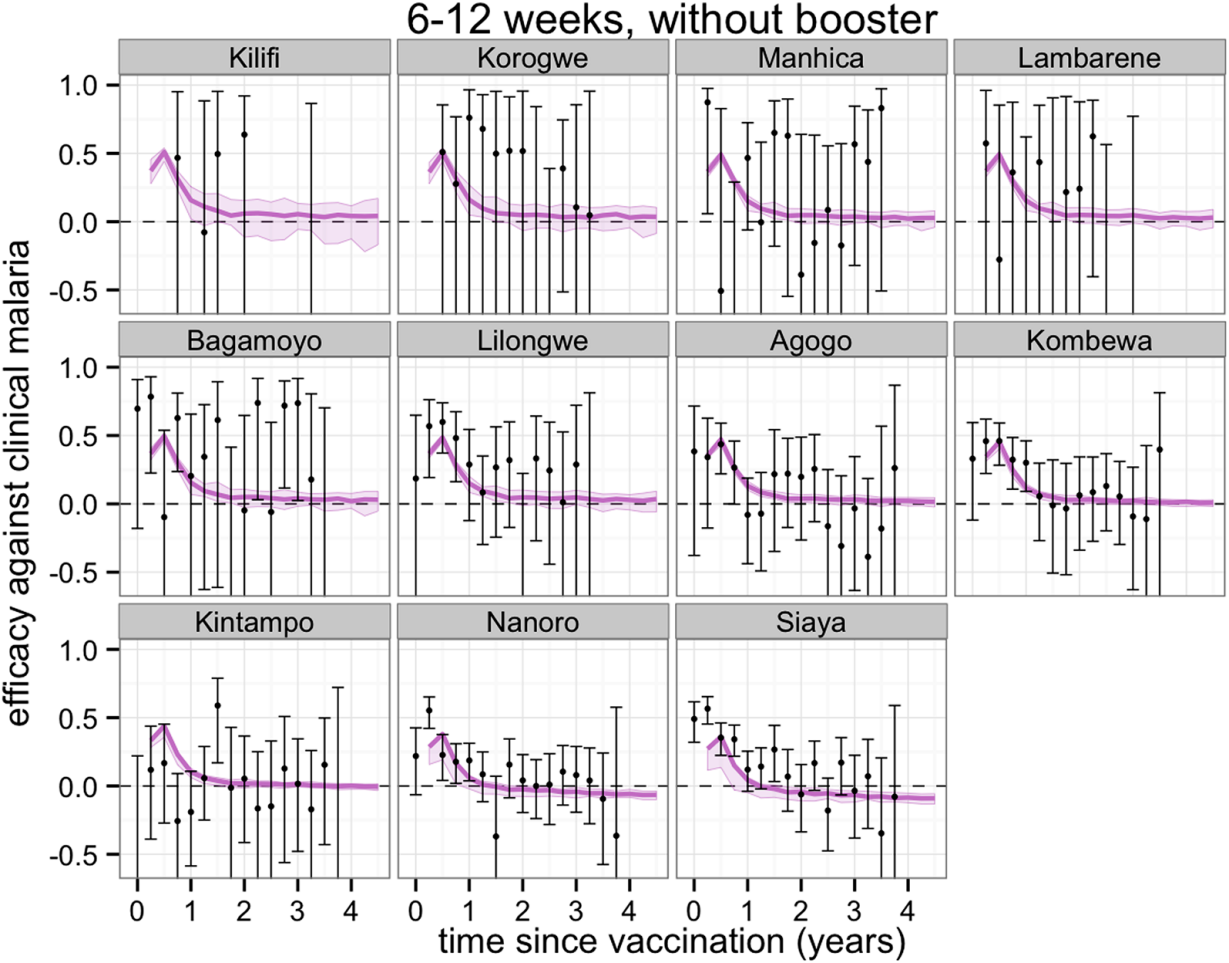
Predicted and observed efficacy against clinical disease by three-monthly periods by trial site for the 6–12 weeks cohort using best-fitted vaccine profile. Reported efficacy (mean and 95 % CI) in the trial site is indicated by *black circles*. Prediction estimates in *purple* (median and 95 % CI)

**Fig. 6:**
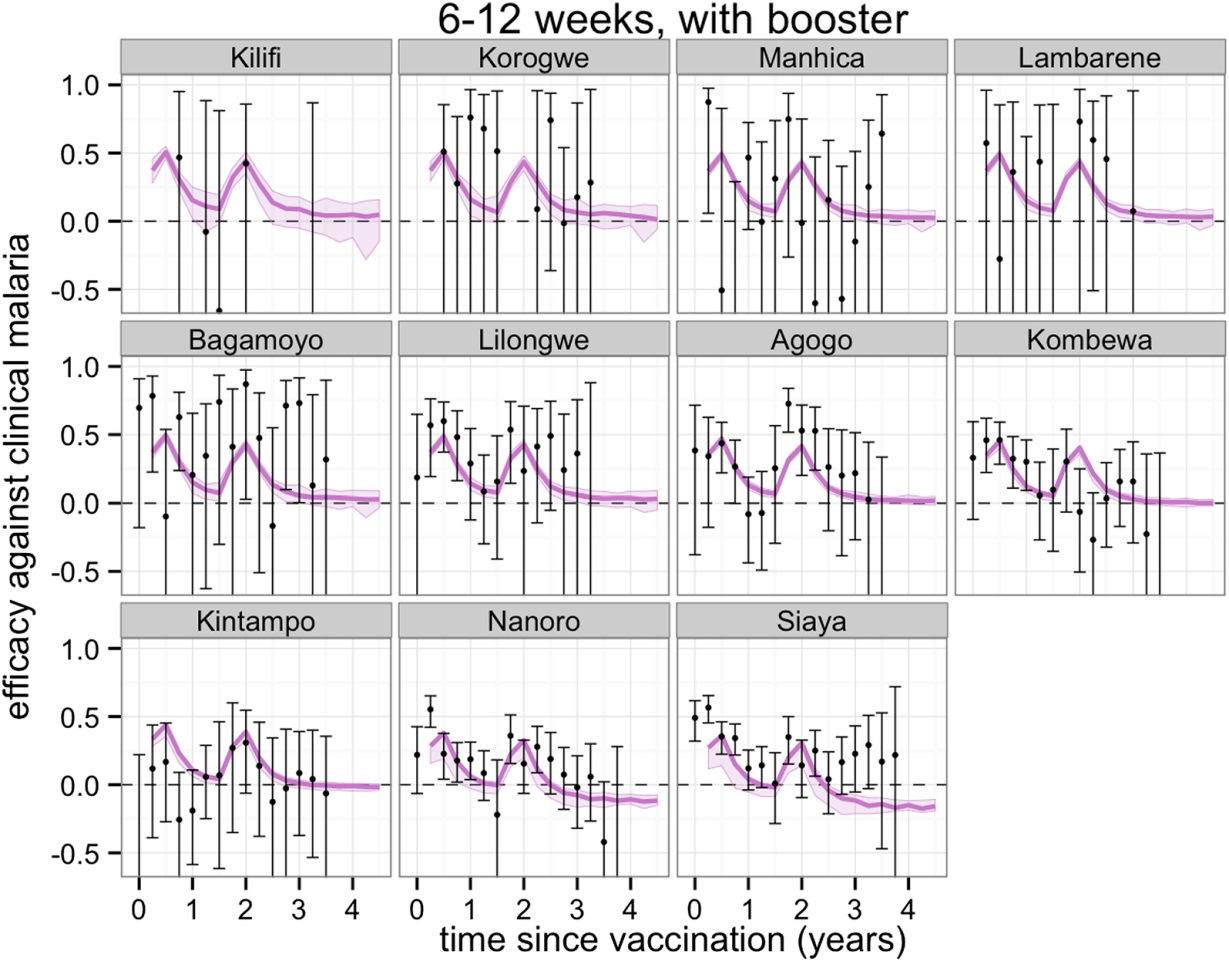
Predicted and observed efficacy against clinical disease by three-monthly periods by trial site for the 6–12 weeks cohort with booster using best-fitted vaccine profile. Reported efficacy (mean and 95 % CI) in the trial site is indicated by *black circles*. Prediction estimates in *purple* (median and 95 % CI)

Figures 3, 4, 5 and 6 show projections of the efficacy against clinical disease over the time periods of the trial follow-up and beyond the course of the trial (longer follow-up than shown in Fig. 2), based on the best-fitting vaccine profile (assuming Weibull decay function). Efficacy against clinical disease decays more rapidly than efficacy against infection. This is because the reduced build-up of natural blood-stage immunity resulting from protection against infection has the effect of delaying, rather than averting some of the clinical incidence in the vaccine arm. There is no such age shifting of infection events because these models assume natural pre-erythrocytic immunity to be negligible. By 3 years after the start of the trial, the efficacy against infection in the 5–17 months cohort is predicted to be close to zero, irrespective of whether a booster dose is included in the schedule (Fig. 4).

**Fig. 7:**
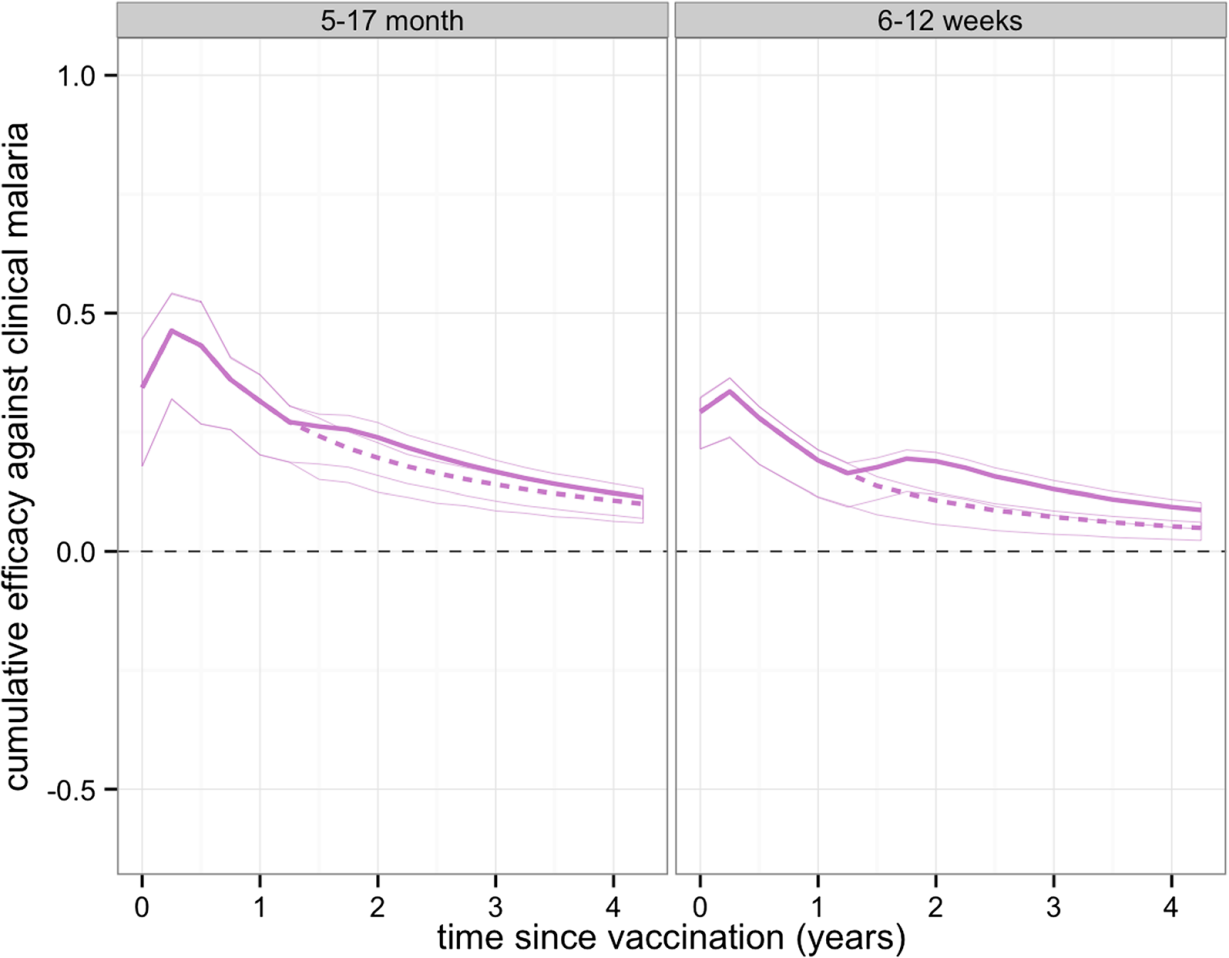
Predicted cumulative vaccine efficacy against clinical disease over time (in years post dose 3) over all trial sites for the **a** 5–17 months cohort and **b** 6–12 weeks cohort with and without booster. The *solid lines* show the predicted efficacy with the booster schedule and the *dashed lines* the predicted efficacy with the non-booster schedule

Overall protection against clinical disease at any time point following the primary course is predicted to be positive over all trial sites (Fig. 7) and potentially close to zero at very long follow-up for the two sites with highest transmission, Siaya and Nanoro (Fig. 7; Additional file 1: Figures S5, S6) This indicates, despite the potential for age shifting, a sustained benefit over the vaccinated population up to 4 years following the primary course.

## Discussion

Clinical efficacy measured in field trials is a key source of information about the likely benefit of deploying preventive interventions against malaria. Assessment of the impact of intervention programmes also needs to consider other factors, including the disease burden in target groups, the coverage achievable, the extent of protection extended to the rest of the population, and, importantly, both the magnitude and duration of effect. Mathematical models of malaria dynamics can be used to integrate information about all of these factors to determine duration of effects and subsequently the prediction of public health impact.

The RTS,S vaccine is currently the focus of decision-making by WHO, and depending on their recommendation, other stakeholders such as Gavi, the vaccine alliance, and national malaria control programmes will also need to make decisions concerning possible implementation. Such evidence-based policy decisions about partially protective vaccines need to take into account both trial data and what is known about malaria epidemiology and health systems (including vaccination programmes) from other sources. The recently completed Phase III trial of RTS,S has provided a substantial amount of data on its effects on clinical incidence in vaccinated children over a 32 months or longer follow-up period. Several different simulation models have been parameterized to fit the diverse outcomes of many previous field studies of *Plasmodium falciparum* to capture the collective knowledge of malaria epidemiologists about malaria dynamics [8, 9, 20–23]. Other publications describe how these models have been linked with data from other sources on vaccination coverage in endemic countries, and malaria disease burden, to project public health impact of RTS,S vaccination programmes, including its impact on outcomes, notably severe disease and death, for which the trial data taken in isolation were not sufficiently powered to provide robust estimates [6, 24, 25].

All models need to take into account that the primary effect of RTS,S is on infection, not on clinical outcomes. RTS,S acts by stimulating an immune response against the CSP of *Plasmodium falciparum* and potentially preventing an infection [26], and therefore its effects on pathogenesis and clinical incidence are indirect. The observed trial results of incidence in the control and vaccinated arms depend on the antibody and immunity longevity and response, but also on a number of factors, including the health system capacity to treat clinical cases, exposure of children to infectious mosquitoes, as well as the dynamical interplay between immunity acquisition, immunity delay, and subsequent effects on clinical and severe disease burden by age. In spite of these complexities, results of clinical incidence and clinical efficacy from the trial can be used, along with data from other field studies and dynamic malaria models, to infer the underlying protection of the vaccine and thus make extrapolations to population effects if the vaccine is recommended.

In this paper, both the characteristics of the vaccine and characteristics of each trial site, such as exposure and treatment levels, were treated as weighted averages of a large number of model realizations [6]. Bayesian fitting (via MCMC algorithms) of model predictions to observed clinical incidence in the cohorts estimates the weights of the vaccine characteristics. This fitting process considers both the stochasticity in the models and the imprecision in the data for the fitting process and uncertainty around the predictions. As investigated previously [6], the most parsimonious model assumes the extent and duration of the induced protection against infection to be the same in all sites, fits the data well.

In general, the fit to the temporal profiles of incidence in the different sites was good (Additional file 1: Figures S1, S2, S3, S4), with simulated efficacy against clinical disease values close to the values measured in the trial (Figs. 2, 3, 4, 5,6), but in some sites (in particular Lambarene, Lilongwe, Korogwe, and Bagamoyo) there was considerable variation in efficacy between time periods (presumably because of random variation), so the relatively smooth trends in incidence predicted by the models were not observed. The tendency for the observed efficacy against clinical disease to be less in higher transmission sites is explained by the dynamics of immune acquisition against repeated infections, and does not require any site-specific assumptions about how the vaccine works.

The results here indicate that following the third dose the initial protection from RTS,S is close to complete in the 5–17 months cohort (91.1 % with the maximum possible value of 100 %), but substantially less than that in infants 6–12 weeks with much larger variation (64 %, Fig. 1). The most likely explanation for this age difference would seem to be that maternal immunity contributes to protection in infants, but that it has no synergistic effect with RTS,S, so that by averting some of the infections that would have been prevented by RTS,S, such as those with lower numbers of sporozoites, it leads to a lower efficacy against clinical disease that can be unambiguously attributed to the vaccine. Immune interference due to administration of RTS,S/AS01 at the same time as EPI vaccines could hypothetically also have an effect [2]. Despite high initial protection against infection in children, the protection in both groups waned quickly, leading to quick waning in efficacy against clinical disease. The decay in efficacy against clinical malaria is due to both decay of immunity for protection against sporozoites and acquisition of natural immunity against clinical disease in the control cohort.

The half-life of decay of efficacy against infection was very similar for the two age groups, but while the fitted curve was similar to an exponential decay in the infants (Fig. 2a), a Weibull decay function with fast decay, followed by longer decay, fits better with the 5–17 months old cohort (assessed via Deviance Information Criterion for nested models, as done previously [6] ). This corresponded to higher efficacy against infection and against clinical disease (Fig. 3) over most of the follow-up period for the older cohort compared to the infants 6–12 weeks (Fig. 5).

The underlying protection gained by a single boosting dose 18 months following the primary course was similar for both cohorts, at around 50 %, but not as high as the initial protection from the final dose of the primary course. This suggests that immune memory cells have shorter duration than normal [27]. The current analysis here assumes that the likely decay of the additional dose is similar for the boosting dose to that for the primary course.

Projections of impact towards the end of the trial and beyond indicate that in low transmission sites the protection against clinical disease is continued. For higher transmission, low clinical efficacy and some age-shifting of clinical incidence is predicted, with such shifting partially observed in the 4-year follow-up of one Phase II trial of RTS,S [28]. Overall, a net positive benefit is predicted up to 4 years after the trial starts, despite some age shifting of events in higher transmission sites. Following any positive WHO recommendation, vaccination of age groups outside those considered in the trial and vaccination covering larger proportions of the population in low transmission settings, will be worth considering to assess indirect effects on transmission and possible local interruption of transmission [29].

## Conclusion

As previously shown [6, 30] and confirmed here with comprehensive analysis of the latest trial results, protection against infection by RTS,S is initially high, but decays quickly and results in a moderately efficacious vaccine against malaria clinical disease [2]. Despite being moderately efficacious, there is much potential for RTS,S to play a role in averting disease and protecting young children most at risk [6]. A recommendation on the use of RTS,S is pending and will need to consider how the underlying protection of the vaccine against infection translates into averted disease morbidity and mortality in more diverse transmission settings and populations than the trial. Models have been carefully calibrated to historical data, and recently RTS,S vaccine action within these models has been rigorously calibrated given availability of trial data to modelling groups [2]. The careful and rigorous calibration of underlying effect of RTS,S sets a high standard for the use of models in future decision making on interventions against malaria (and other infectious diseases), and is essential for sound quantitative prediction of public health burden and cost effectiveness. However, uncertainty in such predictions is unavoidable, especially for more downstream events such as severe disease and mortality. For these outcomes there are only limited calibration data available, if at all, and varying incidence and efficacy patterns across the trial sites is likely due to variation driven by local factors, such as patterns of co-morbidities.

## Additional file

10.1186/s12936-015-0969-8 This file includes additional Figures of results that support and expand some of the results in the main text, but whose inclusion would detract from the main argument.
