# Supplementary material for: The time-course of protection of the RTS,S vaccine against malaria infections and clinical disease
Source: Malar J. 2015 Nov 4;14:437. doi: 10.1186/s12936-015-0969-8 (PMC4634589; doi:10.1186/s12936-015-0969-8)
Supplement: Supplementary file 1 — 10.1186/s12936-015-0969-8 This file includes additional Figures of results that support and expand some of the results in the main text, but whose inclusion would detract from the main argument. [file 12936_2015_969_MOESM1_ESM.pdf]

Additional File 1: Results

The time-course of protection of the RTS,S vaccine against malaria infections and clinical disease

Melissa A. Penny*[1,2], Peter Pemberton-Ross[1,2], Thomas A. Smith[1,2]

* Correspondence: melissa.penny@unibas.ch

1 Department of Epidemiology and Public Health, Swiss Tropical and Public Health Institute, 4051, Basel, Switzerland

2 University of Basel, Petersplatz 1, Basel, Switzerland

This Additional file includes additional results that support and expand some of the results in the main text, but whose inclusion would detract from the main argument.

Tables of Results

Table S1 Best-fitted vaccine efficacy profiles fitting to 6 month data for the 6-12 week and 5-17 months cohort. Posterior distributions described by mean and 95% credible interval

	**Initial efficacy against infection at completion of 3rd dose (%)**	**Half-life of efficacy against infection (months)**	**Decay (weibull decay shape parameter)**	**Boosting efficacy against infection at 4th dose (%)**
**Exponential decay**				
6-12 weeks	55.2 (95% C.I. 39 to 67.9)	7.08 (95% C.I. 6 to 9.24)	-	43.1 (95% C.I. 31.3 to 62.2)
5-17 months	72.7 (95% C.I. 46.2 to 87.3)	8.28 (95% C.I. 6.12 to 20.76)	-	42.4 (95% C.I. 30.8 to 61.7)
**Weibull decay**				
6-12 weeks	57.6 (95% C.I. 40.7 to 72)	7.08 (95% C.I. 6 to 9.36)	0.91 (95% C.I. 0.76 to 1)	44.9 (95% C.I. 31.1 to 66
5-17 months	84.8 (95% C.I. 68.2 to 97.8)	7.2 (95% C.I. 6 to 9.48)	0.76 (95% C.I. 0.59 to 0.96)	46.1 (95% C.I. 30.8 to 68.1)

Figures

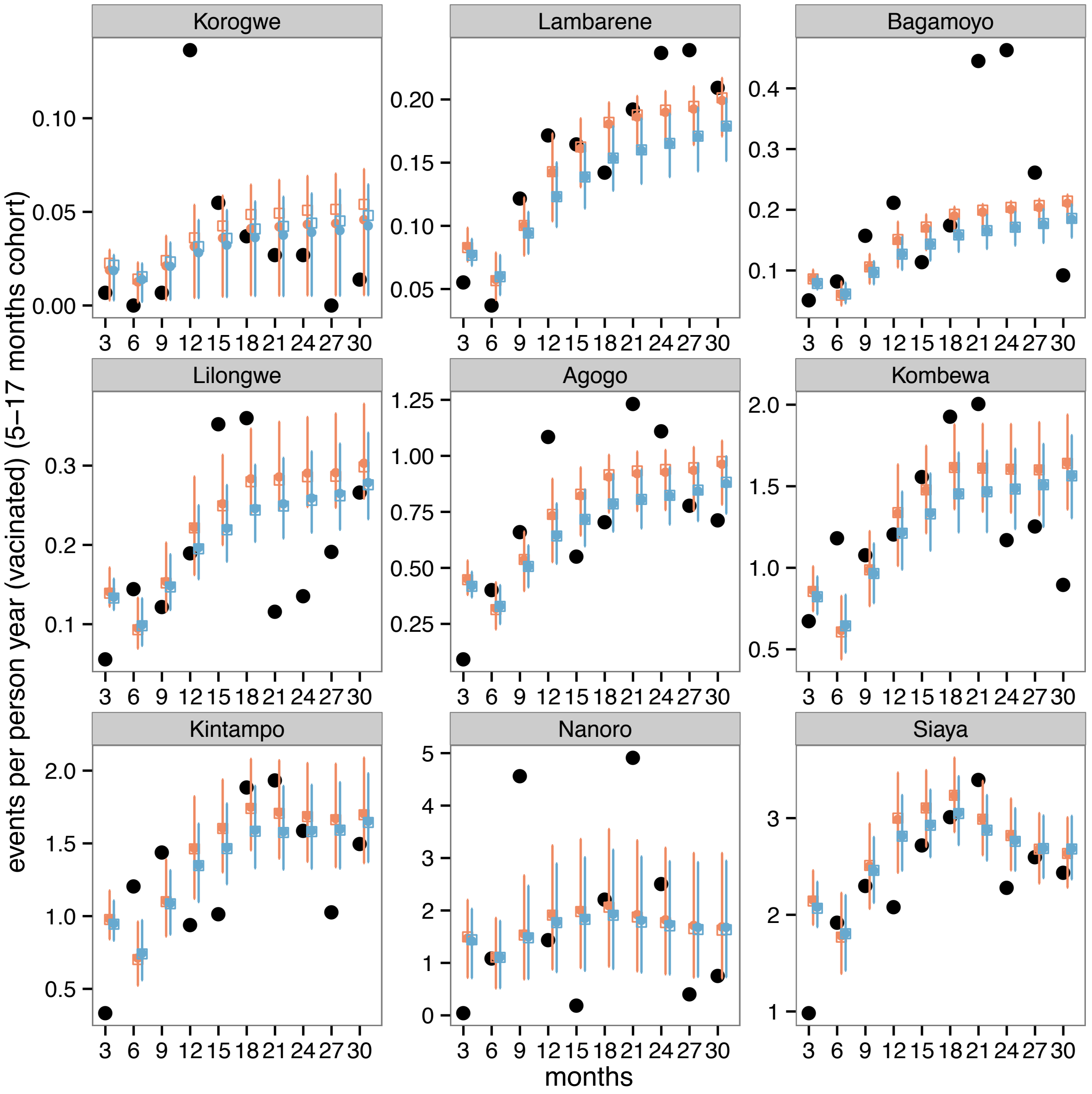

Figure S1 Incidence observed and predicted for 3 monthly periods for vaccinated cohorts. Field and predicted estimates of vaccinated incidence at each 3 month follow-up for the 6-12 weeks cohort by trial site used in the fitting. Mean reported incidence in the trial sites are indicated by black circle. Prediction estimates (mean and 95% C.I.) are shown in colour for different fitted models, orange assuming exponential decay and blue fitting for decay shape.

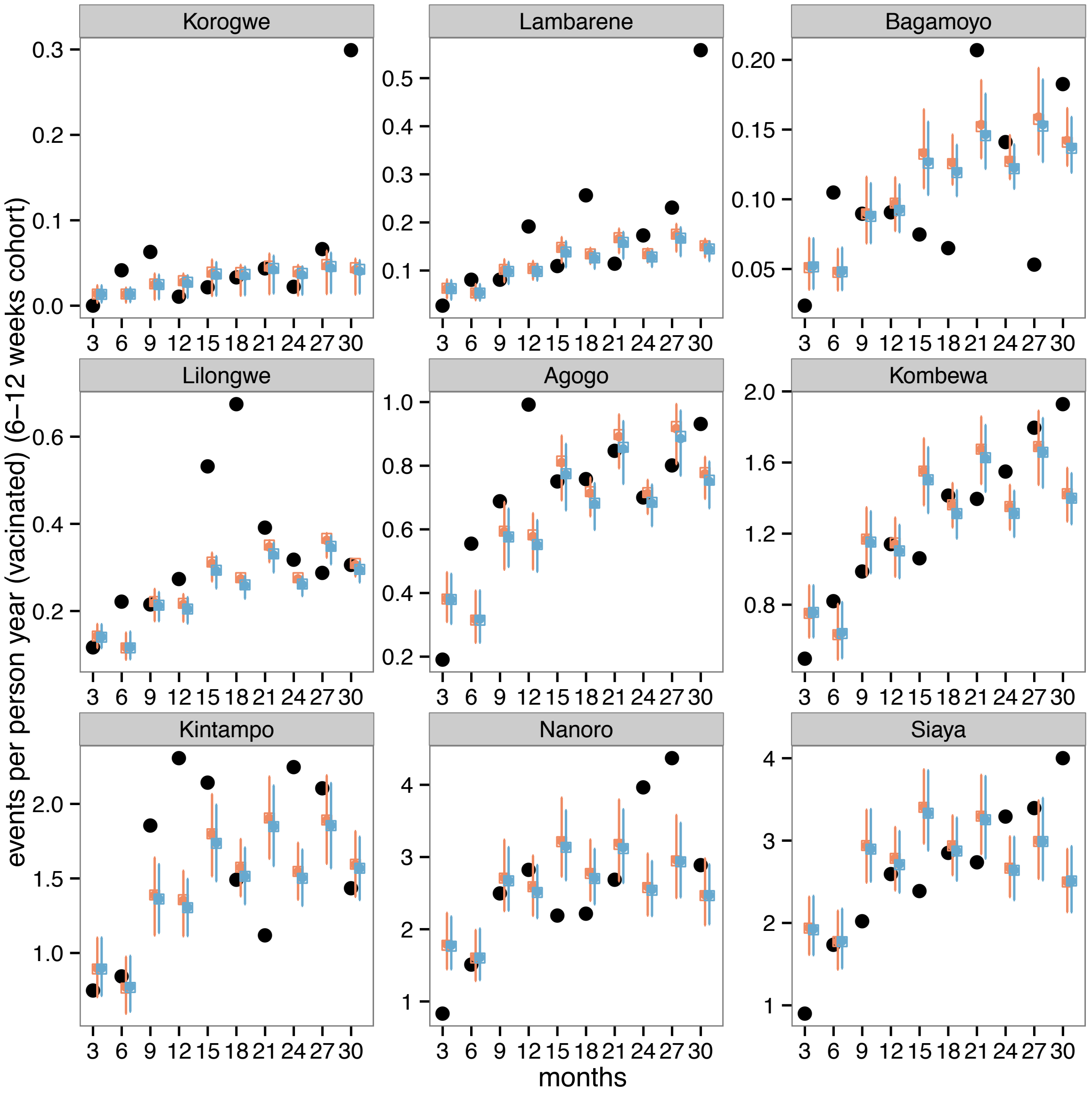

Figure S2 Incidence observed and predicted for 3 monthly periods for vaccinated cohorts. Field and predicted estimates of vaccinated incidence at each 3 month follow-up for the 5-17 months cohort by trial site used in the fitting. Mean reported incidence in the trial sites are indicated by black circle. Prediction estimates (mean and 95% C.I.) are shown in colour for different fitted models, orange assuming exponential decay and blue fitting for decay shape.

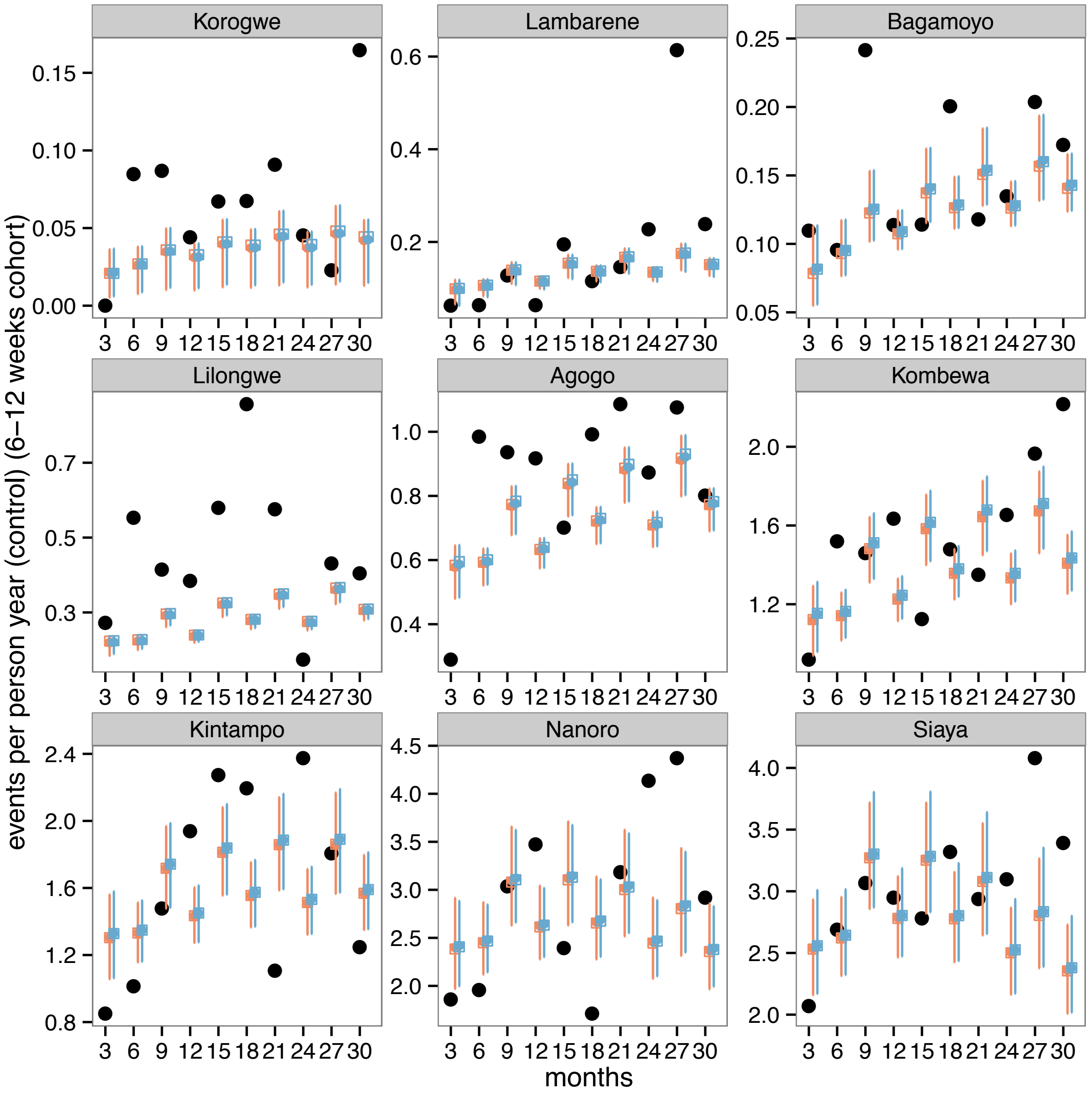

Figure S3 Incidence observed and predicted for 3 monthly periods for control cohorts. Field and predicted estimates of vaccinated incidence at each 3 month follow-up for the 6-12 weeks cohort by trial site used in the fitting. Mean reported incidence in the trial sites are indicated by black circle. Prediction estimates (mean and 95% C.I.) are shown in colour for different fitted models, orange assuming exponential decay and blue fitting for decay shape.

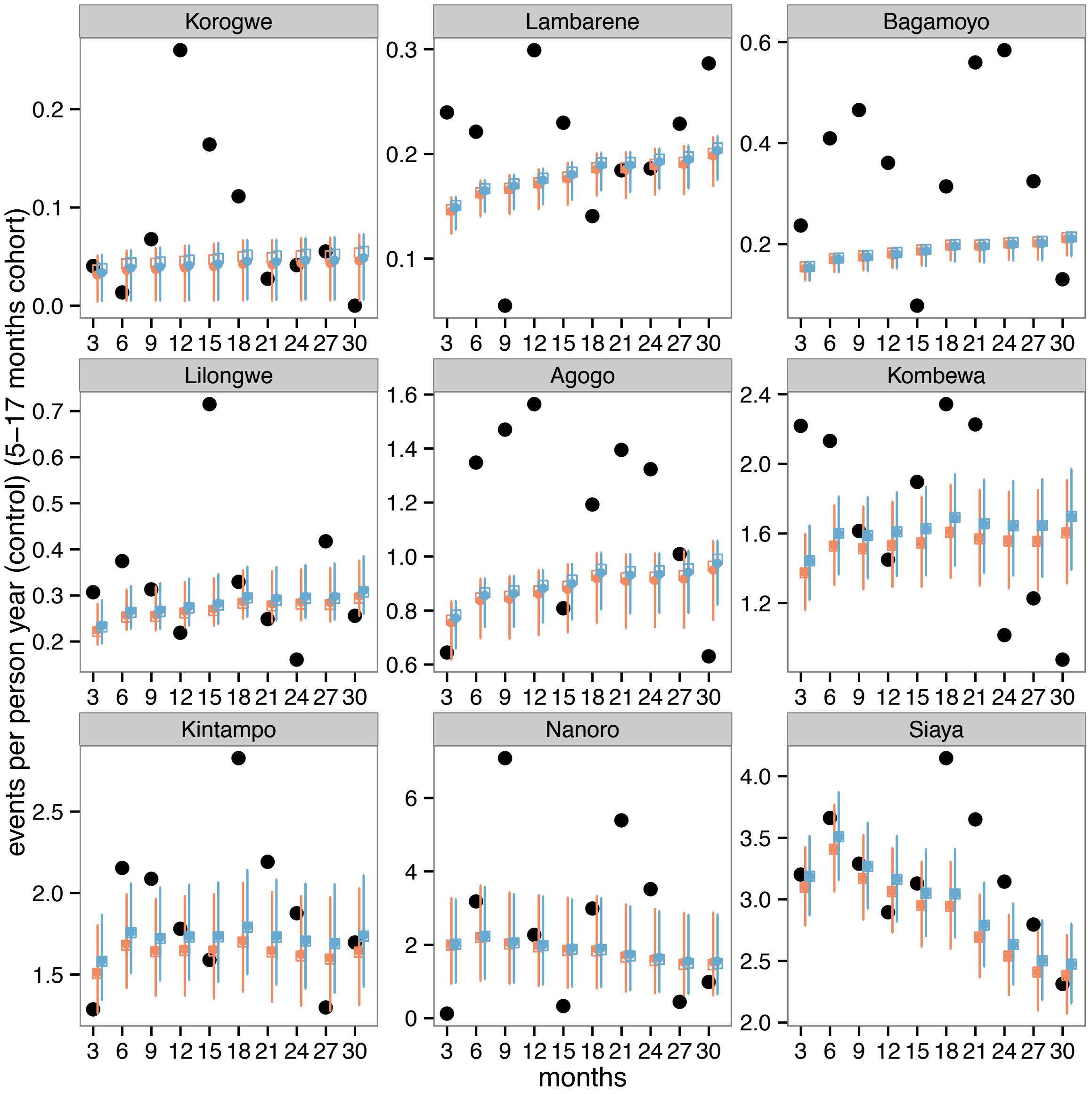

Figure S4 Incidence observed and predicted for 3 monthly periods, for the control cohorts. Field and predicted estimates of vaccinated incidence at each 3 month follow-up for the 5-17 months cohort by trial site used in the fitting. Mean reported incidence in the trial sites are indicated by black circle. Prediction estimates (mean and 95% C.I.) are shown in colour for different fitted models, orange assuming exponential decay and blue fitting for decay shape.

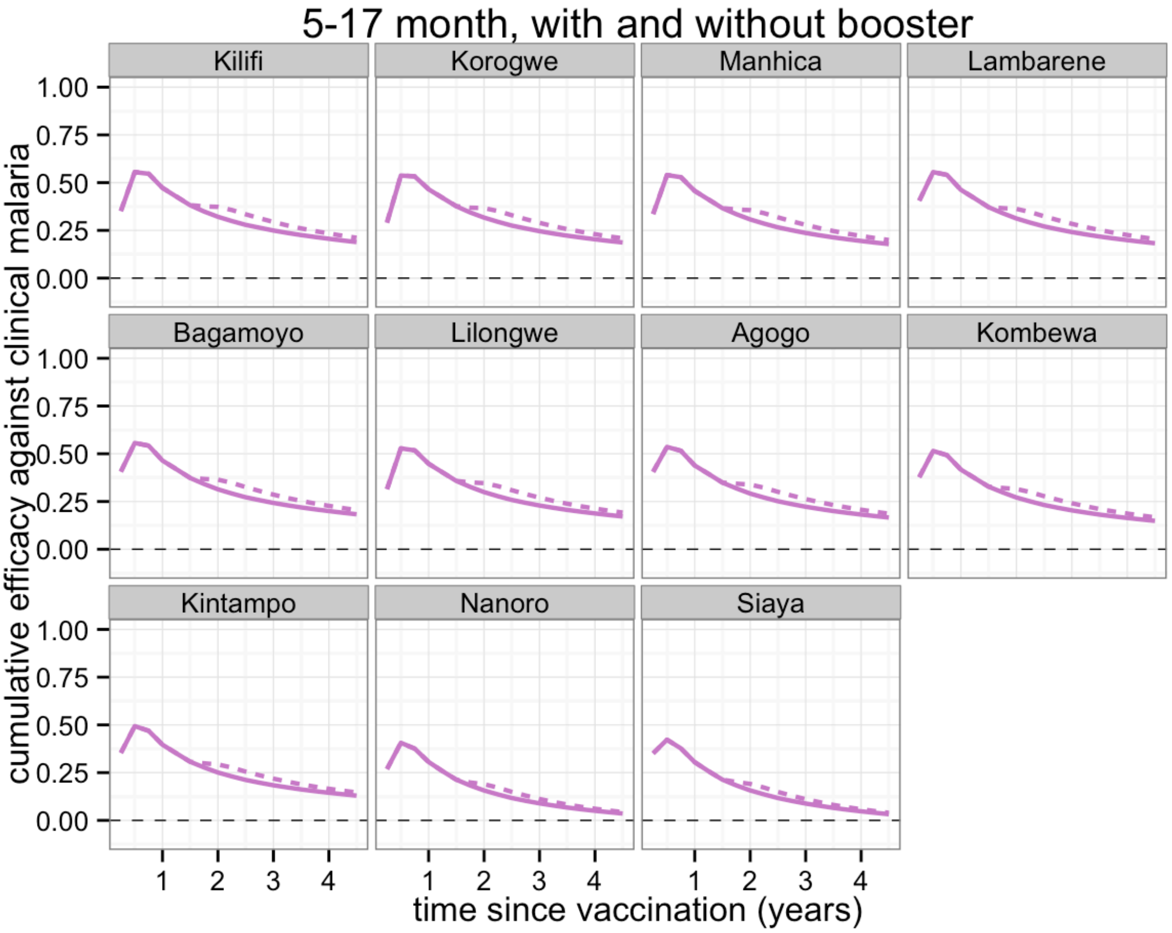

Figure S5 Predicted cumulative vaccine efficacy against clinical disease over time (in years post dose 3) by trial site for the 5-17 months cohort with and without booster. The solid lines show the predicted efficacy with the non-booster schedule and the dashed lines the predicted efficacy with the booster schedule.

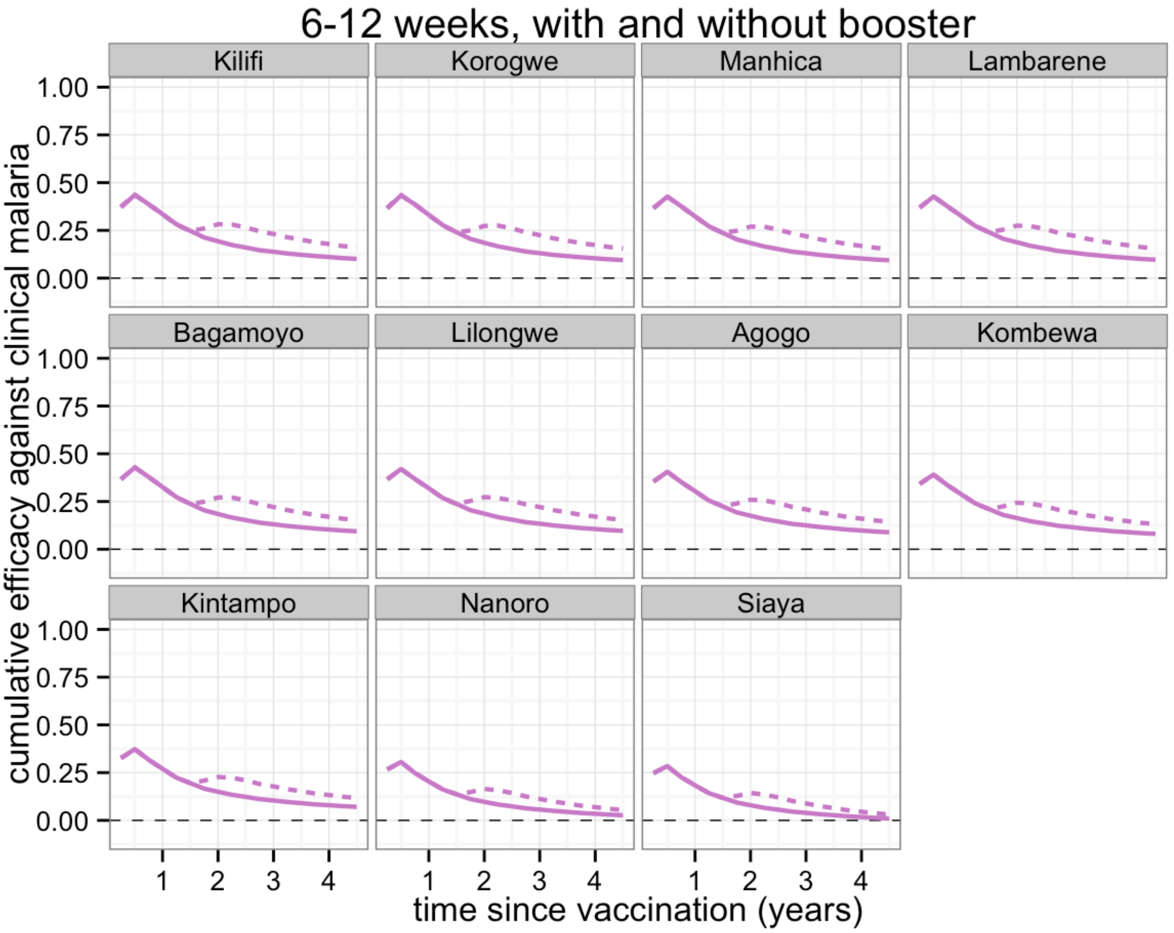

Figure S6 Predicted cumulative vaccine efficacy against clinical disease over time (in years post dose 3) by trial site for the 6-12 weeks cohort with and without booster. The solid lines show the predicted efficacy with the non-booster schedule and the dashed lines the predicted efficacy with the booster schedule.
